# Strength and durability enhancement of low carbon gel concrete

**DOI:** 10.1371/journal.pone.0324319

**Published:** 2025-06-03

**Authors:** Yang Jia, Jianfeng Zhang, Ya Cao, Shiqi Liu, Zichen Zhou, Mingxia Li

**Affiliations:** 1 Wuhan Research Institute of Metallurgical Construction, MCC, Wuhan, China; 2 Changjiang River Scientific Research Institute, Wuhan, China; Mirpur University of Science and Technology, PAKISTAN

## Abstract

In order to reduce the difficulty of concrete preparation, reduce the environmental hazards caused by concrete materials and their preparation process, and meet the development trend of energy saving and emission reduction, this study proposes a low-carbon gel material composed of slag powder, fly ash and gypsum for concrete preparation. Low-carbon gel concrete was made with the ingredients of slag powder, fly ash and gypsum with a water-cement ratio of 0.38 and a ratio of slag powder to fly ash of 1:1.The compressive, flexural and tensile strengths of low-carbon gel concrete samples with different ratios were tested by standard concrete strength test methods to assess their durability properties, and the effects of different ratios of slag powder, admixture and gypsum on the material properties of low-carbon gel were analyzed. concrete material properties with different proportions of slag powder, admixtures and gypsum were analyzed. The experimental results showed that low-carbon gel concrete materials outperformed traditional concrete materials in terms of compressive strength, flexural strength, and tensile strength. After 28 days of curing, the compressive strength of low-carbon gel concrete was 20% higher than that of conventional concrete, and the carbonation depth was reduced by 30.77%. The results indicate that the low-carbon gel concrete materials proposed in this study have excellent strength and durability, and can improve the performance of conventional concrete. This material provides a green and sustainable solution for the modern construction industry, contributing to the sustainable development of the construction sector and the global fight against climate change.

## Introduction

Cementitious materials (CM) are actually substances that can be transformed from a slurry or semi-solid substance into a hard and mechanically strong solid after physical and chemical reactions of the substance itself [1–2]. In modern society, CM has been used to construct many famous buildings, which are obtained by utilizing CM. Proper mixing of coarse and fine particles in the CM system can result in integrated and coordinated development of early and late hardening strength. Long-term continuous hydration ensures the self-healing of initially invisible defects, and a minimum amount of water can be obtained to enable the net slurry to have good rheological properties [3]. Modern CM, on the other hand, is poorly graded and contains a greater number of ultrafine particles, which ultimately increases the quantity of water needed for the configuration, speeds up the rate of hydration, and results in poorly compatible concrete that is not compatible with additives [4]. At present, the cement industry has three main requirements for cement clinker, which are high fineness, high strength and 3CaO·SiO_2_ (C3S). Sulfur trioxide shortage is currently a major issue with concrete that is available on the market, which can negatively affect concrete’s overall performance [5]. The process of cement clinker production correspondingly requires high-temperature calcination. According to data, cement clinker emits a high level of greenhouse gases globally each year, accounting for about 10 percent of the total greenhouse gases. This leads to a gradual warming of the global climate. For this reason, society and international organizations are looking to the construction industry to reduce emissions.

A. M. Tahwia et al. addressed the problem of high carbon emission and resource waste of conventional concrete by proposing the use of proposed partial replacement of ordinary silicate cement by using ceramic slag powder, glass powder, and granite slag powder for the preparation of low carbon high strength concrete [6]. Moreover, for the problem of limited application of polymer composites in engineering geology, they further proposed partial replacement using aluminum-silica enriched fly ash, granite slag powder and volcanic pumice powder [7]. Although existing studies have utilized industrial by-products such as fly ash and slag as alternative CMs, and these materials have good activity and environmental friendliness, their early strength development is slow. In addition, new low-carbon CMs such as limestone may face challenges in raw material supply and quality control for large-scale applications. China, as a major infrastructure construction country, has a high demand for cementitious building materials. Therefore, the experiment innovatively proposes to study low-carbon gel materials (LGM) and analyze the durability and strength of low-carbon gel concrete materials (LGCM), which is expected to grow into a significant avenue for the future development of CM. significant avenue for the future development of CM.

## 1. Composition and properties of low carbon gel materials

### 1.1 Compositional analysis of low carbon gel materials

The study firstly carried out the mastic sand experiments according to the conventional rules of preparing concrete, and systematically studied the composition of CM, while optimizing the dosage of SO_3_. Secondly, a CM system with lower water requirement is designed to meet the required strength and rheological properties of C30 ~ C60 concrete, and the CM system is further optimized. At the same time to carry out different CM composition of the mastic sand strength experiments, which in order to shorten the cracking time and shrinkage time, the experiment based on the results of existing experiments, using clinker coarse grinding, mineral admixture fine grinding method [8–9]. The current standard cement mastic sand water cement ratio is 0.5, which is very different from the basic principles of concrete production. Therefore, the experiment evaluates the quality of CM according to the basic principles of concrete production. At the same time, in order to meet the requirements, the study utilizes the experimental method of special glue sand to test the strength of LGM’s glue sand, based on the commonly used concrete’s water-to-cement ratio (0.30 ~ 0.46), and the experiment takes the middle value of 0.38 as the water-to-cement ratio of special glue sand [10–11]. Among them, the information related to raw materials slag powder and fly ash is shown in Table 1.

**Table 1:** Physical, chemical and rheological properties of slag powder and fly ash.

Characterisation	Slag powder	Fly ash
Particle size distribution (μm)	10-50	5-20
Specific surface area (m^2^/kg)	573.0	450.0
Density (g/cm^3^)	3.1	2.3
Water absorption (%)	1.5	2.0
SiO_2_ (%)	35.0	50.0
Al_2_O_3_ (%)	15.0	20.0
Fe_2_O_3_ (%)	10.0	5.0
CaO (%)	30.0	15.0
Viscosity (Pa·s)	1.2	0.8

Based on the raw material characterization information shown in Table 1, the specific design of the test method is finally obtained as follows: First, the water-cement ratio (WCR) is set to 0.38. The water reducer (WR) dosage amount is adjusted to ensure that the flowability of the cement mortar on the jumping table can reach 200 mm ~ 220 mm. Secondly, according to GB/T17671-2021 *Testing Method of Cementitious Sand Strength* (ISO), the qualified standard specimen specimen is made as 40 mm × 40 mm × 160 mm, and the relevant specimen is maintained under the standard curing conditions in order to reach the corresponding age, and then the compressive and flexural strength (FS) of rubber sand specimen are tested according to GB/T17671-2021. Table 2 displays the rubber sand CM composition design.

**Table 2:** Composition design of LGM in rubber sand testing.

Number	Clinker/%	Slag powder/%	Fly ash/%	SO_3_/%
X1	50.0	50.0	/	3.0
X2	50.0	50.0		4.0
X3	40.0	60.0		3.0
X4	40.0	60.0		4.0
X5	30.0	70.0		3.0
X6	30.0	70.0		4.0
X7	20.0	80.0		3.0
X8	20.0	80.0		4.0
Y1	50.0	/	50.0	3.0
Y2	40.0		60.0	3.0
Y3	30.0		70.0	3.0
Z1	50.0	10.0	40.0	3.5
Z2	50.0	20.0	30.0	3.5
Z3	50.0	30.0	20.0	3.5
Z4	40.0	20.0	40.0	3.5
Z5	40.0	30.0	30.0	3.5
Z6	40.0	40.0	20.0	4.0
Z7	30.0	50.0	20.0	4.0
Z8	30.0	45.0	25.0	4.0
Z9	30.0	40.0	30.0	4.0
Z10	20.0	60.0	20.0	4.5
Z11	20.0	50.0	30.0	4.0
Z12	20.0	40.0	40.0	3.5
Z13	20.0	30.0	50.0	3.5

**Note:** Gypsum is mixed according to the SO_3_ content conversion, slag powder (SP) is used (mobility ratio of 106%) with a specific surface area (SSA) of 573m^2^/kg, fly ash (FA) is class III ash (water requirement ratio of 109%), and gypsum is used with flue gas desulphurization gypsum, which has an SO_3_ content of 41.66%.

In Table 2, from the X system, it can be found that when the SO_3_ content is increased from 3% to 4%, the 28d strength of the collodion sand exhibits a significant increase and is generally increased by 3−4 MPa, which indicates that the moderate use and increase of SO_3_ can contribute to the strength of the collodion sand material. In order to further classify the CM, the experiments refer to the studies of existing scholars to screen and clarify the quality of SP, the selection range of WCR, and the grade of FA in low clinker CM [12–13]. In addition to the four sets of concrete mixing ratios selected through the experiment, several additional sets of ratios have been established and are mainly represented by two commonly used concrete strength grades of C30 and C50, and the design of mixing ratios is based on the commonly used mixing ratios of the current mixing plant. A reasonable range of water-cement ratio is determined with reference to the existing concrete mix design specification (GB/T 17671−2021 “Method for testing the strength of cementitious sand”) and empirical data from relevant literature, and according to the strength class of concrete (C30, C50) and fluidity requirements. Table 3 lists the precise mixing ratios.

**Table 3:** Multiple sets of concrete mix ratios.

Number	Clinker	Slag powder	Fa	Gypsum	Xtone powder	Water	Sand	Stone	Water-binder ratio/ × 10^−2^
C30-X8	67	267	/	36	/	170	833	1017	46
C30-Z8	100	150	84	36					
C30-Z9	100	134	100	36					
C30-Z10	67	200	67	36					
C50-X8	88	350	/	47		155	673	1098	32
	C50-Z8	131	197	110	47				
	C50-Z9	131	176	131	47				
	C50-Z10	88	263	88	47				
C30-A1	100	150	84	36		170	833	1017	46
	C30-A2	67	200	67	36				
	C30-B1	74	162	41	26	67			
	C30-B2	93	148	37	26	66			
C50-A1	131	197	110	47		155	673	1098	32
C50-A2	88	263	88	47					
C50-B1	97	214	53	34	87				
C50-B2	121	194	49	34					

In Table 3, the first eight items are the optimized LGCM ratios of the collodion, and the SP used is from Zouping County Xintai Environmental Protection Building Materials Co. After grinding, the SP SSA is 573m^2^/kg, the 7d activity index is 55%, the 28d activity index is 83%, and the fluidity is 106%. There are two types of SP used in the last eight items, S105 and S95. The LGCM ratios for the SP grade comparison group [14]. The experiments establishes an upper limit of 30% clinker admixture for the low cost LGM technical requirements. In comparison, the current CM system commonly uses in mixing stations sets the FA admixture between 22% to 30%. As indicated in Table 4, the trials also looked at replacing the FA with ultrafine limestone powder (ULP) to get the ideal composition of the low clinker CM.

**Table 4:** Optimized composition of low clinker cementitious material.

Number	Clinker/%	S105/%	FA/%	Plaster/%	Stone powder/%
A-20	20	48	22	10	/
A-24	24	44			
A-28	28	40			
A-32	32	36			
B-20	20	48	16		7
	B-24	24	44		
	B-28	28	40		
	B-32	32	36		

In Table 4, system A represents no addition of stone dust, system B represents addition of stone dust, and 10% gypsum is converted to 4% SO_3_ admixture.

### 1.2 Effect of different gypsum and admixtures on the properties of low carbon gel materials

#### 1.2.1 Effect of additives on the properties of low carbon cementitious material.

**Fig 1:**
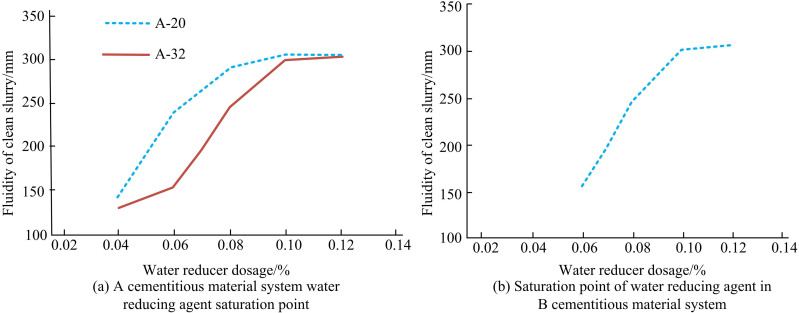
Water reducer saturation point (WRSP) of CM system of A and B.

Modern concrete should be produced in accordance with production requirements, which means that admixtures should be added as part of CM during the production of CM. Moreover, the corresponding CM should be tested in accordance with the basic principles of concrete production [15–16]. The concrete preparation process is as follows: First, CMs such as cement clinker, slag powder, fly ash and gypsum, as well as coarse aggregate, fine aggregate, water and admixture are weighed according to the designed ratios. Second, the CMs are added to the mixer for dry mixing, completely mixed, and the measured water and admixtures are added according to the set water-cement ratio and continuously mixed for 3 minutes. The admixtures used in the experiments are polycarboxylic acid (PCA) powder admixtures, and the variation of the saturation point of the admixtures for the two CM systems A and B is shown in Fig 1.

Fig 1a shows the saturation point of WR for CM system A. The saturation point of WR for CM systems A-20 and A-32 both showed 0.10%. Moreover, under the existing experimental data and conditions, the saturation point of WR would be not significantly affected by the doping of S105SP with the Liangyu ultrafine milled limestone powder. Fig 1b shows the variation of WRSP of CM system of B. Based on the variation of CM saturation point of A, the experiment here only tested the WRSP of CM system B-28 group CM system of B. It can be observed that the WRSP performance of CM system of B is 0.11%.

**Fig 2:**
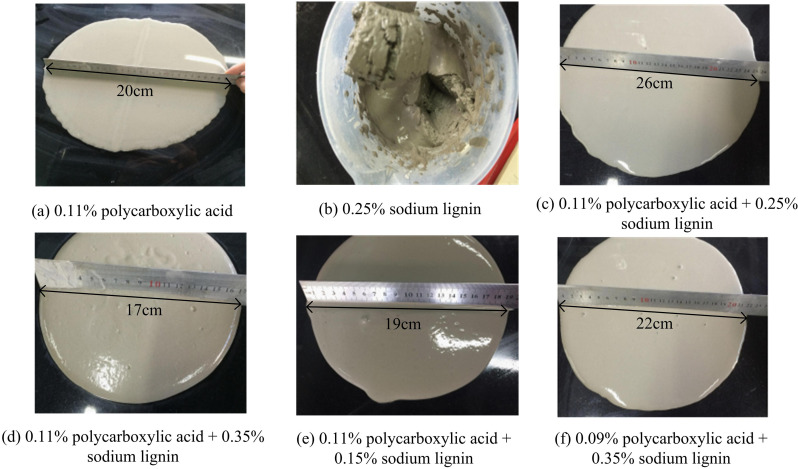
Effect of different types and proportions of admixtures on net slurry and FCP.

To investigate the impact of admixture type on elongation control and finished product quality of LGM, an experiment is conducted to compare the compatibility of naphthalene WR and powder polycarboxylate superplasticizer (PCSP) with LGM [17–18]. Group B-28 LGM system is selected and the experiment is carried out with reference to the saturation point of the admixture, and the compatibility between LGM and the corresponding admixture is judged by detecting the flow of the net slurry at different admixture amounts of different admixtures. The specific experimental program is as follows: (1) Admixture 1: 0.11% PCA + 0.25% WS+(0.11% + 0.25%) (PCA + WS). (2) Admixture 2: 0.15% WS+(0.11% + 0.35%) (PCA + WS). (3) Admixture 3: 0.35% WS+(0.09% + 0.25%) (PCA + WS). (4) Admixture 4: (0.11% + 0.15%) (PCA+sodium wood). The slurry preparation process is as follows: First, all raw materials are strictly screened and pre-treated, and the slurry proportioning scheme is determined according to the experimental design, and the water-cement ratio is set at 0.38. Second, the weighed raw materials are put into the mixer for dry mixing, and water is added according to the set water-cement ratio. Then, mixing is continued for 3 minutes, and a suitable amount of admixture is added in the mixing process to improve the fluidity. Fig 2 illustrates the impact of various additive types and ratios on the fluidity of cement paste (FCP).

In Fig 2, it can be found that when sodium lignite is doped alone, the state of net slurry shows a stiff state and cannot flow. When sodium lignite is superimposed with PCA, the increase in the amount of sodium lignite resulted in a corresponding decrease in the flowability of the net slurry. This indicates that the adaptability between WS and LGM system is not good, and the compatibility between WS and PCSP is not good, and the combination between surface LGM system and PCA additives is better. The addition of sodium lignin did not improve the fluidity of the slurry as other additives did, but instead resulted in larger voids and a hardened state of the slurry. This phenomenon may be related to the poor compatibility between sodium lignin and the LGM system. The molecular structure of sodium lignin as an additive interacts unfavorably with other components in the LGM system (e.g., slag powder, fly ash, etc.), resulting in a decrease in slurry fluidity.

#### 1.2.2 Effect of different gypsum additions on the properties of low carbon cementitious material.

Reasonable application of gypsum in CM system can not only effectively regulate the setting time, but also stimulate the active dopant in CM system and compensate the material shrinkage to improve the anti-cracking performance [19–20]. To investigate the impacts of various gypsum types on the functionality of LGM, three types of gypsum are chosen: anhydrous gypsum (A), gypsum dihydrate (B), and desulfurization gypsum (C). Moreover, group A-24 CMs are selected for the experiments and the amount of gypsum is converted according to 4% of SO_3_ content. In the FCP experiments, the mass of CM used is about 300 g, 87 g of water, and a water-gum ratio of 0.29:1. Table 5 displays the test findings that are achieved.

**Table 5:** Test results of the effect of different types of gypsum on FCP, water consumption, and setting time.

Group	Initial expansion/mm	Expansion after half an hour/mm	Expansion loss/mm for half an hour
A	190	180	10
B	200	180	20
C	260	245	15
Group	Standard consistency water consumption/%	Initial setting/min	Final setting/min
A	24.6	223	310
B	24.2	225	322
C	23.1	335	380

From Table 5, it can be seen that the initial extensibility of gypsum in groups A and B is much smaller than that of group C. Anhydrous gypsum group A has the smallest value of initial extensibility, which indicates that desulfurization of gypsum is more conducive to the water-demanding qualities of the LGM system. After half an hour of expansion, Group A gypsum had the least loss of expansion, Group B gypsum had the greatest loss of expansion, and Group C gypsum had the middle loss of expansion. Overall, anhydrous gypsum (A) has a high water requirement and low swelling loss due to its dense chemical structure. While gypsum dihydrate (B) has a lower water requirement and higher swelling loss due to the presence of water of crystallization. Desulfurized gypsum (C), on the other hand, had a lower water requirement and moderate swelling loss due to its good dispersibility and hydration activity, and longer initial and final setting times, giving it the best overall performance. Meanwhile, the chemical interaction of SO3 in the concrete matrix promotes the formation of calcite and accelerates the hydration process. Meanwhile, improving the pore structure and optimizing the interfacial transition zones improved the strength and durability of the concrete. Comprehensive analysis of the FCP and the results of the water consumption for consistency shows that in order to ensure that the LGM system meets the necessary water demand, the experimental use of desulfurization gypsum for the preparation of LGM system is the most appropriate.

## 2. Strength and workability of use of concrete with low carbon gel materials

### 2.1 Experimental design program

Based on the above analysis, under the premise that the SP activity meets the experimental requirements, the clinker content of not less than 20% can also be configured to meet the concrete strength requirements of concrete [21–22]. At the same time, the economic and experimental objectives are comprehensively considered, and four clinker mixing LGMs of 20%, 24%, 28% and 32% are designed to be obtained. To carry out relevant experimental research on concrete, two regularly used grades, C30 and C50, are set up. The test follows the standard: *GB/T 50080−2016 Standard Test Method for Properties of Ordinary Concrete Mixes, GB/T 50081−2019 Standard Test Method for Mechanical Properties of Ordinary Concrete, GB/T 50082−2009 Standard Test Method for Long-Term Properties and Durability of Ordinary Concrete.* Table 6 displays the test mix ratios for C30 and C50 LGM in relation to the benchmark concrete group.

**Table 6:** C30, C50H low carbon cementitious material concrete and the base group of concrete test mix ratio (kg/m^3^).

Number	Clinker	Slag powder	Fly ash	Gypsum	Stone powder	Water	Fine aggregate	Coarse aggregate	Admixture
C30-A-20	74	178	81	37	/	155	833	1017	0.11%
C30-A-24	89	163							
C30-A-28	104	148							
C30-A-32	118	133							
C30-B-20	74	178	56		26				
	C30-B-24	89	163						
	C30-B-28	104	148						
	C30-B-32	118	133						
Number	Cement	S95	FA	Gypsum	Stone powder	Water	Fine aggregate	Coarse aggregate	Admixture
C30-J	220	60	90	/		170	833	1017	2.0%
C50-J	280	100	105			155	673	1098	2.2%
Number	Clinker	Slag powder	Fly ash	Gypsum	Stone powder	Water	Fine aggregate	Coarse aggregate	Admixture
C50-A-20	97	233	107	49	/	146	673	1098	0.18%
C50-A-24	116	213							
C50-A-28	136	194							
C50-A-32	155	175							
C50-B-20	97	233	73		34				
	C50-B-24	116	213						
	C50-B-28	136	194						
	C50-B-32	155	175						

Note: The additives used in C30-J and C50-J systems are liquid PCSP, and the dosage of additives is based on the actual. The system with A represents no stone powder, and the system with B represents stone powder. 20, 24, 28 and 32 correspond to the clinker content, and the additives use powder PCSP, and the dosage of additives is based on the actual.

In Table 6, in LGCM, the WCR of C30 and C50 concrete is 0.42 and 0.30, using Class II FA, ULP SSA of 650 m2/kg, SP level of S105, and powder PCSP as admixture. In addition, the WCR of conventional cementitious materials (CCM) and concrete mix ratio is 28% with SP level of S195, and 33.00% with liquid PCSP as admixture. In addition, in the CCM and concrete mixing ratio, SP grade S195 is used, liquid PCSP is used as admixture, the water reduction rate is 28%, and the solids content is 33.00%.

### 2.2 Harmony analysis of concrete

The workability of concrete mainly includes fluidity, water retention and cohesion [23]. The experiment firstly analyzes the fluidity of concrete, in which slump and extension are important indexes to characterize the fluidity of concrete. And Table 7 displays the particular outcomes.

**Table 7:** Comparison of the flow ability of low carbon cementitious material with the baseline group of concrete mixes.

Number	Slump/mm	Expansion/mm	Number	Slump/mm	Expansion/mm
C30-J	190	/	C50-J	200	/
C30-A-20	210		C30-B-20	210	
C30-A-24	200		C30-B-24	210	
C30-A-28	200		C30-B-28	210	
C30-A-32	190		C30-B-32	230	480
C50-A-20	250	500	C50-B-20	270	550
C50-A-24	200	/	C50-B-24	250	520
C50-A-28	200		C50-B-28	250	520
C50-A-32	190		C50-B-32	240	500

**Fig 3:**
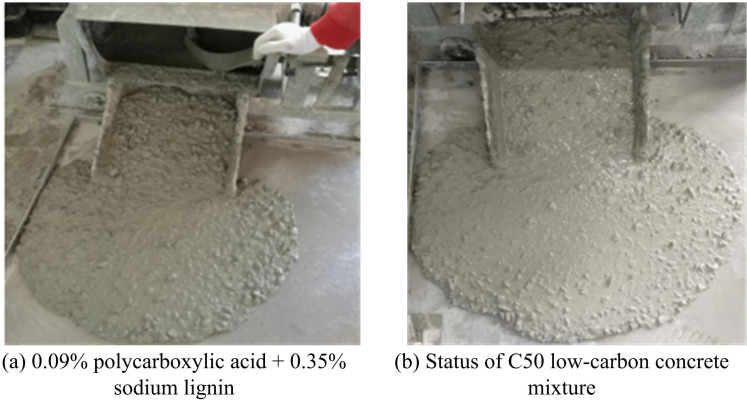
State of two low carbon concrete mixes C30 and C50.

Table 7 shows that the low clinker CM group’s concrete has a far bigger slump than the benchmark group’s, with all of the slump sizes being greater than 180 mm. This represents that the prepared LGM has reached the S4 or S5 grade, which meets the requirements for large flowability concrete. In addition, the proportion of S105-SP started to decrease under the increase of clinker proportion, and the slump showed a decreasing trend. This is because S105-SP has a flowability of up to 106%, and when the mass is replaced equally, the paste’s volume increases and the concrete’s fluidity is enhanced. Because ULP makes the concrete more flowable, the water demand of LGM is also substantially lower than that of CCM and may satisfy the requirements. The state of the two low carbon concrete mixes, C30 and C50, is shown in Fig 3.

Fig 3 displays the state of the concrete out of the pan. The two types of concrete equipped do not have the phenomenon of segregation and water secretion, and the cohesion of the concrete is better, and the encapsulation of water is good.

### 2.3 Analysis of compressive, flexural, and tensile strength of concrete

#### 2.3.1 Effect of different clinker admixture on compressive strength (CS) of concrete.

**Fig 4:**
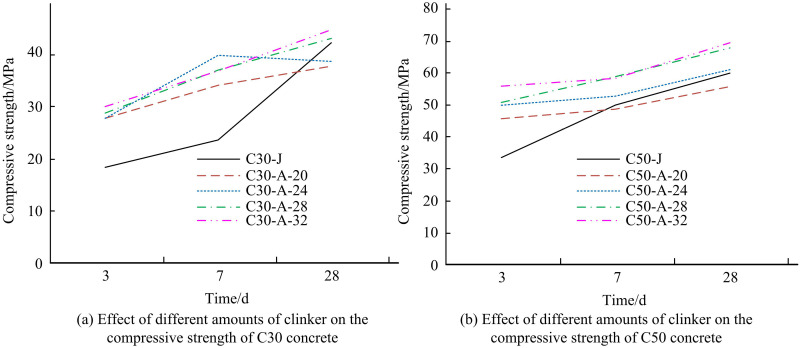
Effect of different clinker admixture on CS of concrete.

The variation of CS of C30 and C50 concrete with age for different clinker admixtures is shown in Fig 4.

Fig 4a shows the effect of different clinker admixture on the CS of C30 concrete. With the increase of clinker admixture in CM, the CS of C30 concrete at different curing ages (CAs) are gradually increased. The strength of C30-A grade concrete is close to 30.00 MPa at 3 d. In addition, the strength at 28 d has a maximum value of 44.62 MPa at a clinker admixture of 32%. The impact of various clinker admixtures on the CS of C50 concrete is displayed in Fig 4b. The CS of C50 concrete is much improved along with the rise in clinker consumption in CM. The strength of C50-A grade concrete tends to be 50.00 MPa at 3d. In addition, the CS at 28d has a maximum value of 69.02 MPa when the clinker mixing amount is 32%. Except for the experimental group with clinker mixing amount of 20.00% and S105-SP mixing amount of 48% which has a smaller CS at 28d. All the rest of the C30 and C50 concretes can well all the remaining C30 and C50 concretes can well meet the needs of experimental and actual engineering on the development of early CS of concrete. This is mainly because the reduction of the WCR improves the CS of concrete with low clinker CM system. At the same time, the more active S105-SP can better compensate for the early defects of high mineral, low clinker and CM system. The above results show that in the low clinker integrated CM system even if the clinker admixture is reduced to 24%, the prepared concrete still meets the required CS of concrete in the project.

**Fig 5:**
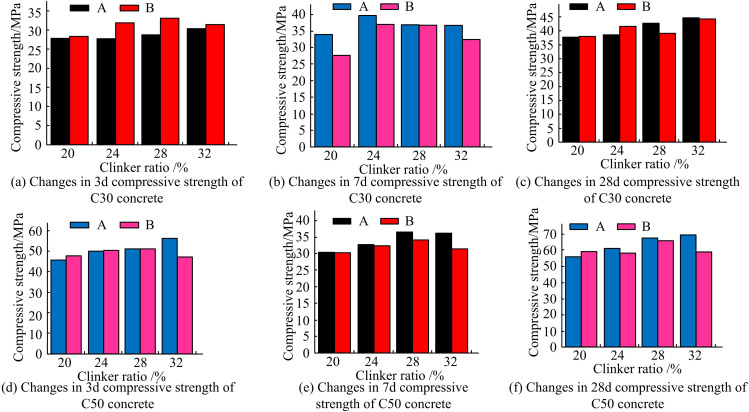
Effect of ultrafine limestone powder on CS of concrete.

The use of suitable ULP can reduce the amount of water used in concrete mixes [24]. For this reason experiments are conducted on ULP to continue wringing and grinding to a SSA of 650 m2/kg, and as well as the mix ratio to replace FA with 7% ULP in equal quantities, to analyze its effect on the strength of low carbon integrated CM concrete. Fig 5 illustrates how ULP affects the CS of concrete.

**Fig 6:**
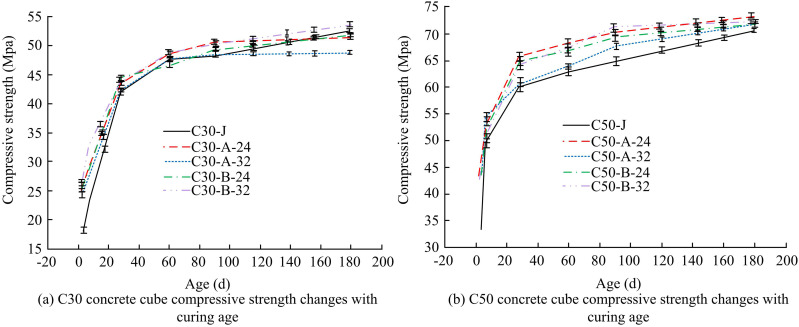
Variation of CS of C30 and C50 concrete cubes with CA.

Fig 5a–5c all show the effect of ULP on the CS of C30 concrete. In Fig 5a, when the CA reaches 3d, the CS of C30 concrete of system B is all stronger than that of CM of system A. When the clinker admixture is 24%, the strength of concrete of system B is higher than that of the material of system A by 4.41 MPa. This also shows that the appropriate addition of a certain amount of ULP is able to increase the 3d CS of the low clinker CM concrete without affecting the 28d CS. This may be due to the fact that ULP can reduce the nucleation site barriers and accelerate the hydration reaction process of CM, which ultimately improves the early CS of LGM [25]. In Fig 5b, the CS of CMC30 concrete of system A is greater than that of system B at 7d. And in the clinker mixing amount of 20%, the gap between the two systems of concrete strength is larger, but still can meet the strength requirements of low clinker CM system concrete use. At 28d, the CS of each group of concrete can also meet the requirements. Fig 5d–5f show the effect of ULP on the CS of C50 concrete, respectively. The CSs of C50 concrete prepared by LGM of system A concrete and that prepared by CM of system B are very similar at 3d, 7d and 28d CAs. This indicates that %ULP equivalent replacement of FA does not have an unfavorable effect on the early strength development of low clinker C50 strength grade concrete. Overall, the optimum value of concrete varies for different number of days of curing and for different clinker contents. This may be due to the fact that concrete with higher clinker content has higher strength in early and long curing, while concrete with lower clinker content gradually approaches or even exceeds the strength of high clinker concrete in medium and long curing due to the continuous hydration reaction of reactive admixtures. Fig 6 illustrates the change in cubic CS of C30 and C50 concrete with CA.

The cubic CS change curve of C30 concrete with CA is displayed in Fig 6a. All ages of C30 concrete meet the requirements for strength development, and all ages of the low clinker group’s concrete have generally higher strengths than the benchmark group. There is a slight rise in the 3d CS of C30 concrete following the addition of 7% ULP to replace the FA, and the strength remains unaffected after 28d. The change curve of C50 concrete’s cubic CS with CA is displayed in Fig 6b. In addition, the strength of C50 concrete meets the requirements for strength development at different ages. When FA is replaced by 7% ULP in the same amount, the strength of the concrete remains unchanged at all ages, with the strength of the low clinker group being higher than that of the currently used CM group. Fig 6 shows that the current concrete strength has reached 180d of age with normal strength development. This indicates that the strengths of the concrete in the low clinker group all meet the requirements and are generally higher than those of the benchmark group. The strength of concrete is not harmed by substituting 7% ULP for FA. In fact, it can even strengthen C30 concrete in 3D.

#### 2.3.2 Flexural and tensile strength of concrete.

**Fig 7:**
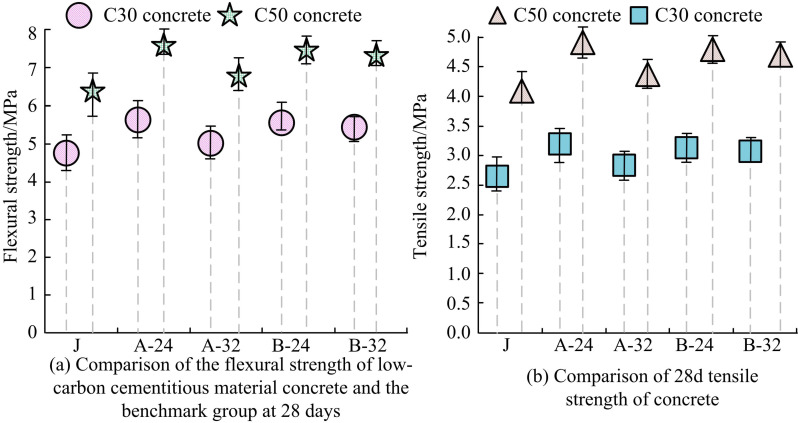
Comparison of flexural and tensile strength of concrete.

Three groups of benchmark group, clinker group 24% and clinker group 28% are selected for the experiments on the FS and tensile strength (TS) of concrete. Fig 7 presents the concrete findings.

The 28d FS of LGCM is compared in Fig 7a. A maximum of almost 1 MPa is shown in the FS of the C30 and C50 concrete groups, all of which exceed that of the benchmark group. This suggests that adding ULP can somewhat enhance the FS of concrete. When the proportion of clinker reaches 32%, the FS of C30 concrete increases to a maximum of 0.4 MPa. Fig 7b shows a comparison of the 28d FS and TS of the concrete, and the FS and TSs of the C30 concrete group are all greater than that of the benchmark group. In the C50 concrete group, the FS and TS of the A-32 group is comparable to that of the CCM system, while the FS and TSs of the other groups are all greater than that of the benchmark group. However, the addition of ULP generally improves the FS and TS of concrete, and can increase up to 25%.

## 3. Durability and microscopic properties of concrete with low carbon gel materials

### 3.1 Experimental design program

In fact, the longevity of the prepared concrete is crucial to the building structure’s safety and correct operation [4]. The experiments are mainly compared with the benchmark group to analyze the durability and microscopic properties of concrete. The durability mainly includes carbonation, permeability and frost resistance. In order to combine to meet the needs of the project in practical applications, the concrete carbonation experiment is designed to study a total of four CAs of 0, 1, 3, and 7d, respectively. The freeze-thaw specimens are 100 mm × 100 mm × 400 mm prisms and the carbonation depth (CD) specimens are 100 mm × 100 mm × 100 mm cubes cured under standard curing conditions for up to 28 days. The environmental conditions for the freeze-thaw test are −15°C to 20°C, and for the CD test are 20°C, 65% relative humidity, and 20% CO_2_ concentration. In addition, the experiments select the clinker proportion of 24% and 32% of the low clinker combination of the benchmark group for comparison experiments.

### 3.2 Durability analysis of concrete

#### 3.2.1 Effect of different CAs on the carbonation depth of concrete.

**Fig 8:**
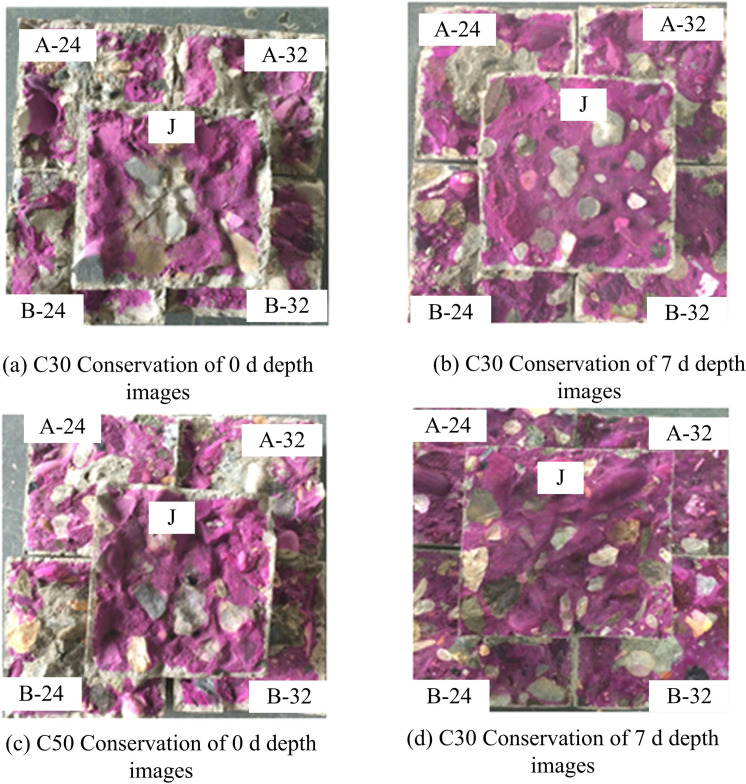
NC images of C50 and C30 concrete with different casting methods.

The surface of concrete structures may peel off due to carbonation in the surrounding natural environment, which can have a major negative impact on the durability performance of those structures [26]. Fig 8 shows C30 and C50 concrete in different natural carbonation (NC) images.

**Fig 9:**
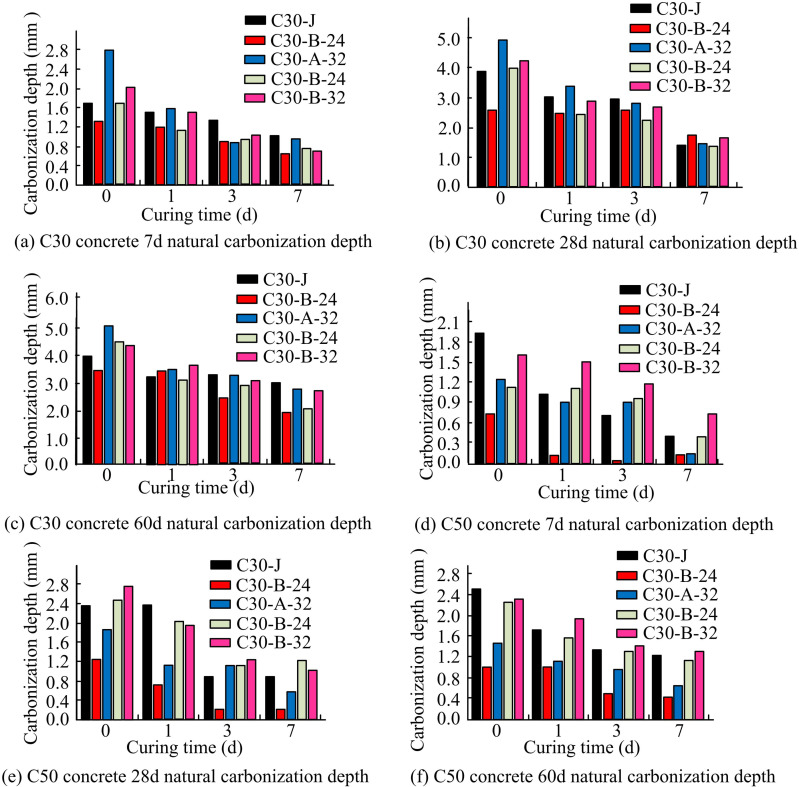
Variation of NCD of C50 and C30 concrete with different CAs.

Based on the results shown in Fig 8, the variation of C30 and C50 concrete at different natural carbonation depths (NCD) is further analysed as shown in Fig 9.

At various CAs, the NCD of C30 concrete is displayed in Fig 9a–9c. It is discovered that as CA has increased, the NCD of concrete has been trending downward. Meanwhile, compared with the CA of 0 and 7d, the decrease in the NCD of C30-J is between 0.6 mm and 2.6 mm, while the CD of concrete with low maturity CM group starts to decrease and is between 1.0 mm and 3.5 mm. This suggests that the CD of the low clinker group of concrete decreases more significantly as the age of curing increases. Fig 9d–9f illustrate the NCD of C50 concrete at varying CA values. When the CA is 0 and 7d, the decrease in the NCD of C50-J concrete ranges from 1.5 to 2.1 mm, while the decrease in the NCD of low clinker group concrete ranges between 0. and 2.6 mm. This means that while the age of curing increases, the NCD of concrete in the low clinker group tends to decrease much more than that of the baseline group. In addition, ULP improves the early and late strength of LGM concrete. It improves the concrete’s resistance to carbonation, seepage and freezing by optimizing the pore structure and increasing hydration products.

**Fig 10:**
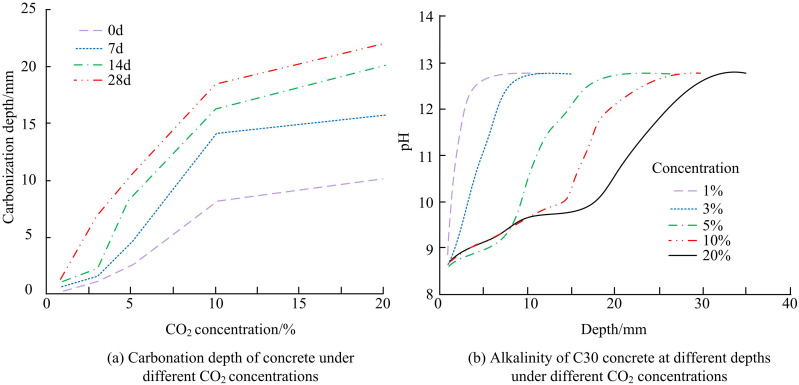
CD compared to alkalinity change for C30 concrete.

The specimens are moulded according to the mixing ratio and then cured under standard conditions until 28 days, followed by accelerated carbonation in 1%, 3%, 5%, 10%, and 20% CO_2_ concentration environments, with carbonation ages of 0, 7, 14, and 28 days, respectively, during which the temperature is maintained at a constant temperature of 20 ± 2°C and relative humidity of 70 ± 5%. C30 concrete cement paste specimens at different depth ranges at standard age (28d) are tested for alkalinity and the results are shown in Fig 10.

Fig 10a illustrates the CD of C30 concrete at various CO2 concentrations. The test results show that the CD of C30 concrete increases as the CO2 concentration rises at the same carbonation age, and that the carbonation rate increases initially before slowing down. Furthermore, under the same CO_2_ concentration, concrete’s CD is rising together with the carbonation age. At the early stage of concrete carbonation, the growth trend of CD is larger. And when the carbonation age reached 28d, the growth trend of concrete carbonation began to slow down. Fig 10b shows the alkalinity of C30 concrete with different depths under different CO_2_ concentrations. Under the condition of carbonation age of 28d, under the same CO_2_ concentration, the alkalinity value of the concrete gradually increases from surface to inside with the increasing CD: the pH value at a certain depth from the outer surface of the concrete has a relatively large change, and the internal pH basically does not change. At the same CD, with the increasing of CO_2_ concentration, the alkalinity in concrete showed a decreasing trend.

#### 3.2.2 Comparison of concrete permeability and sulfate attack resistance.

By comparing the benchmark group, the 28d permeability of low clinker concrete and benchmark group concrete is studied, and the effects of ULP and S105-SP on the 60d permeability of low clinker group concrete are synthesized. The experiments are conducted by the electric flux method, and the evaluation indexes of the electric flux method are as follows: the permeability of chloride ions is higher when the total electrical conductivity is greater than 4000. When the total conductivity is within 2000 ~ 4000, the permeability of chloride ion is in the middle. When the total conductivity is within 1000 ~ 2000, the permeability of chloride ion is low. The permeability of chloride ions is very low when the total conductivity is within 100 ~ 1000. The permeability of chloride ions is negligible when the total conductivity is less than 100 [4,8–28],[2]. Table 8 displays the experimental findings on the permeability of concrete.

**Table 8:** Electrical flux of low carbon cementitious material concrete compared to benchmark group.

Concrete 28d electric flux test results			
Number	Electric flux test value/C	Number	Electric flux test value/C
C30-J	2301	C50-J	1206
C30-A-24	952	C50-A-24	358
C30-A-32	988	C50-A-32	499
C30-B-24	621	C50-B-24	344
C30-B-32	1043	C50-B-32	467
Number	Electric flux test value/C	Number	Electric flux test value/C
C30-J	1321	C50-J	708
C30-A-20	1187	C50-A-20	310
C30-A-24	894	C50-A-24	324
C30-A-28	822	C50-A-28	268
C30-A-32	759	C50-A-32	278
C30-B-20	1048	C50-B-20	269
C30-B-24	550	C50-B-24	262
C30-B-28	811	C50-B-28	284
C30-B-32	723	C50-B-32	299

In Table 8, the 28-day electric flux of LGCM is lower than that of the benchmark group, indicating greater impermeability. As the clinker dosage increases, the electric flux increases and the permeability decreases, which is consistent with the results of the CD test. However, the addition of ULP reduces the electric flux, but the effect is not significant. In the 60-day electric flux test, the electric flux of C30 concrete in the low clinker group is 700C-1400C, which is lower than that of the benchmark group at 1326C. The electric flux of C50 concrete is centered around 250 C, which is lower than that of the benchmark group at 710 C. This indicates that the resistance to chloride penetration of LGCM is better than that of the benchmark group. From 28 days to 60 days, the permeability gap between LGCM and CCM decreases. This is mainly due to the higher activity of the SP used in the experiment at the early stage, which causes the LGCM to hydrate faster and form a denser structure at the early stage, thus improving the permeation resistance. By 60 days, the degree of hydration of the two is close to each other and the difference in properties decreases, which is consistent with the development pattern of concrete for construction [29,30].

**Fig 11:**
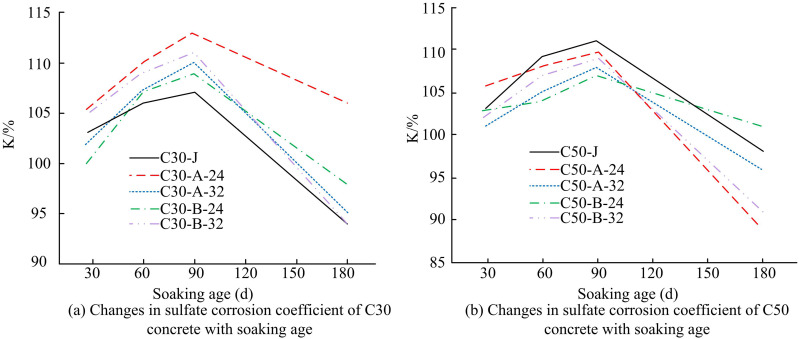
Comparison of concrete resistance to sulfate attack.

The chloride permeability of LGCM still offers a significant advantage over existing high performance concrete (HPC) systems. As stated in reference [31], the 28-day electrical flux of conventional HPC typically ranges from 1500 C to 2000 C. In contrast, the 28-day electrical flux of LGCM is considerably lower than that of conventional HPC. In contrast, the 28-day electrical flux values of LGCM are much lower than this range, indicating that it is superior to existing HPC in terms of resistance to chloride ion permeability. Comparison of concrete resistance to sulfate attack is shown in Fig 11.

Fig 11a and 11b show the variation of sulfate erosion coefficients with immersion age for C30 and C50 concrete, respectively. As the immersion time of concrete in sulfate solution increases, its resistance to sulfate attack shows an upward trend, which reflects that concrete specimens immersed in solution for a long period of time have higher strengths compared to those immersed in water only. This phenomenon can be attributed to the crystallization of sulfate in the porous structure of the concrete, which promotes densification, whereas the crystals formed in the process did not generate sufficient expansion forces to cause structural damage. Thus, this increased densification contributes to the compressive capacity of the concrete. However, this increase in strength is not sustained, and when the immersion time reaches the node of 180 d, the coefficient of resistance to sulfate attack (K-value) is generally below 100, indicating that the internal structure of the concrete specimens began to suffer damage, leading to a reduction in strength. In the overall trend, the K-values of LGM-made concrete showed little difference or a slight increase compared to the control group.

#### 3.2.3 Analysis of concrete frost resistance.

**Fig 12:**
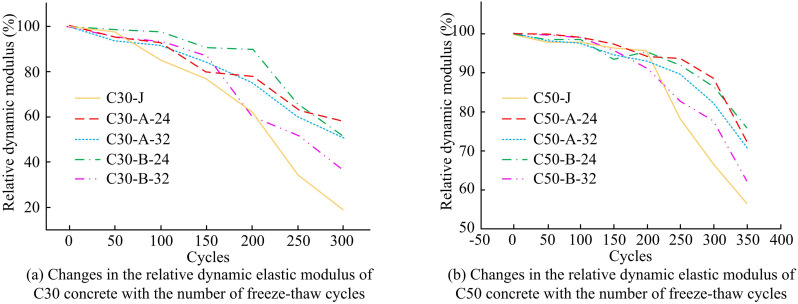
Variation of relative dynamic modulus of elasticity with the number of freeze-thaw cycles for different concretes.

Experiments are conducted to evaluate the frost resistance of concrete since concrete structural buildings are exposed to extremely cold weather throughout the winter. In Fig 12, the relationship between the number of freeze-thaw cycles (FTCs) and the relative dynamic modulus of elasticity (RDME) of C30 and C50 concrete is displayed.

Fig 12a shows the variation of RDME of C30 concrete with FTCs. The decreasing trend of RDME obtained for C30 concrete starts to become very steep at the end of the 150th FTC and the internal structure of the concrete is severely damaged from this point onwards. Even if the RDME of the C30-J and C30-B-32 groups is less than 60% when the number of FTCs approaches 250, the latter group’s RDME is significantly greater than the benchmark group’s. However, at this time, the RDME of all the other concrete groups is greater than 60%. Overall, the RDME of LGM is superior and the CM concrete with 24% clinker addition has better frost resistance. Fig 12b shows the variation of RDME with the number of FTCs for C50 concrete. When the number of FTCs reaches 200, the decreasing trend of RDME of concrete starts to become steeper and the internal structure of concrete starts to be damaged at this time. Furthermore, all of the LGCM group’s relative dynamic elasticity objectives are much greater than those of the benchmark group, suggesting that the low clinker group’s concrete has significantly better frost resistance than the benchmark group’s.

### 3.3 Microscopic properties of low-carbon gel materials

**Fig 13:**
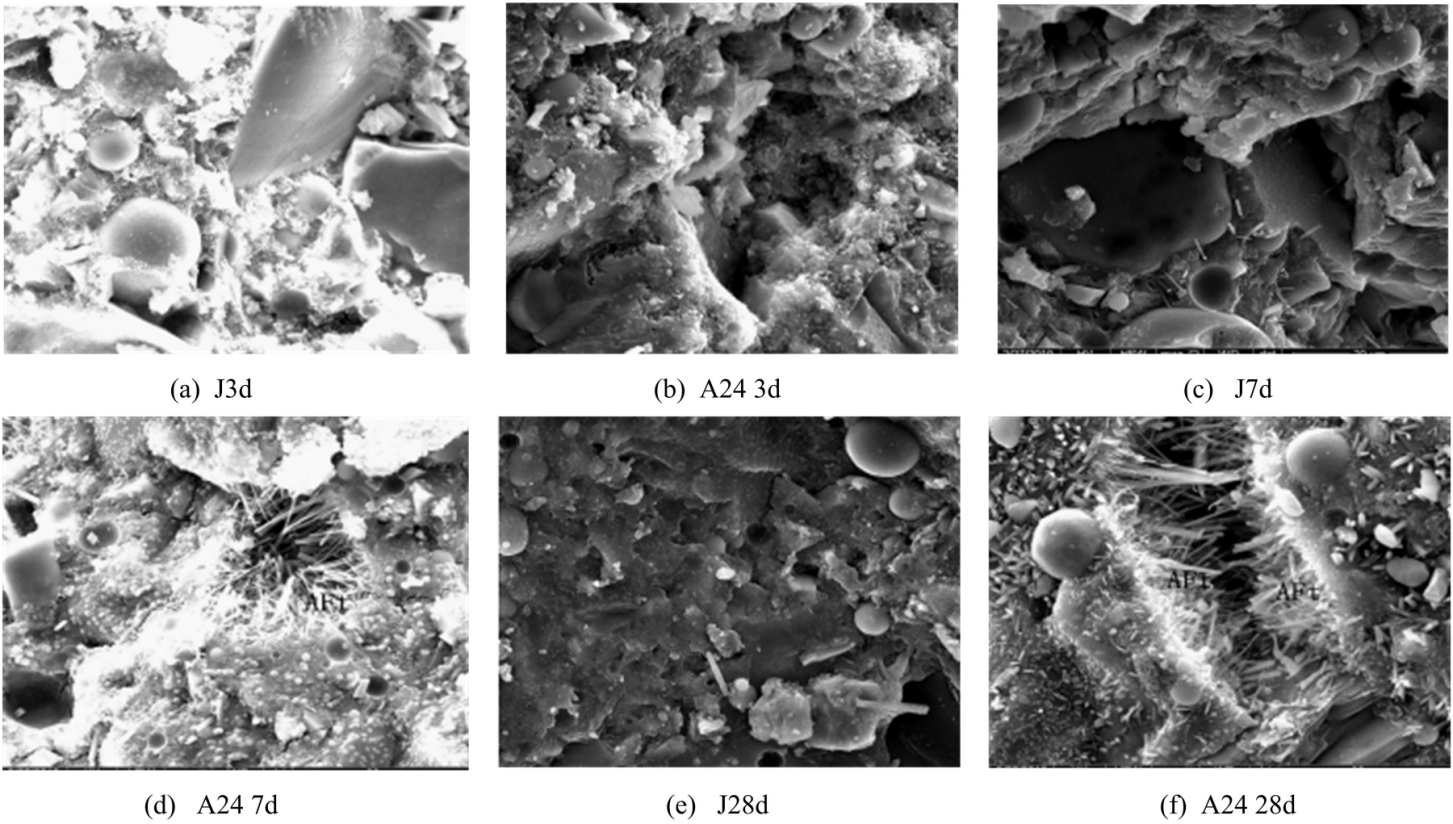
SEM images of low carbon cementitious material and conventional cementitious material net slurry specimens.

Changes in time and temperature lead to changes in the microstructure of concrete, which also implies that concrete is highly dynamic in nature, as well as highly non-homogeneous [32]. The scanning electron microscope (SEM) image obtained by magnifying the net paste specimen of LGCM by 5000 times under the microscope is shown in Fig 13.

In Fig 13, the WCR of the net concrete mortar specimens used for the experiments are all 0.38. It can be seen that when the net mortar experiments of the benchmark group of CM systems reaches the CA of 28d, all of them produces a hydrated gel except for a small amount of FA particles that did not react. However, the LGM group still has a large amount of FA that do not undergo hydration as well as other more unhydrated particles at 28d. This also indicates that the hydration reaction of the benchmark group (Group J) is basically completed at 28d, which may lead to the insufficient CS and inverted shrinkage of the commonly used CM system concrete at the later stage, but the LGM do not have such a problem.

In addition, comparing the J group with the LGM group, it can be found that in the SEM images of the reference group (J group), there is no alumina in the net slurry at different maintenance ages, and no alumina has been found in the LGM group when the maintenance age reaches the 3rd d. However, when the age of the LGM system reaches 7d, calcite begins to appear in the LGM system. These calcium alumina materials have the potential to both partially raise and decrease the susceptibility of concrete buildings to cracking. In summary, the LGM microstructure is denser and less porous. There are more calcite and C-A-S-H gels in its structure, which results in higher stiffness and helps to increase the modulus of elasticity of the material and reduce the erosion of the material by sulfates.

## 4. Environmental benefits assessment

The study quantified the environmental benefits of LGM over its entire life cycle using the life cycle assessment (LCA) methodology and analyzed them in comparison to existing low-carbon cement technologies. Details are presented in Table 9.

**Table 9:** Comparison of energy savings and emission reductions per tonne of material for different cement technologies.

Method	CO_2_ reduction (t)	Energy consumption (GJ)
CCM	0.80	3.50
LGM	0.47	2.75
Renewable energy cement	0.62	2.95
Carbon capture and storage technology	0.28	3.45
Blast furnace slag cement	0.53	2.48
FA cement	0.58	2.65

In Table 9, LGM performs better in reducing CO2 emissions and energy consumption, outperforming traditional cement materials and other common low-carbon technologies. LGM uses slag powder and fly ash to replace part of the cement clinker. It not only reduces carbon emissions but also reduces energy consumption in the production process. The SP production process consumes less energy, only about 1/3 of cement clinker, while FA is a by-product of coal-fired power plants with good filler properties and some activity. The LGM technology outlined in the study has the potential to yield positive environmental impacts by partially replacing cement clinker with industrial by-products such as SP and FA.

According to the market price of cement materials in China, the price of cement clinker is 350 RMB/t, the price of SP is 150 RMB/t, and the price of FA is 100 RMB/t. The raw material cost of LGM can be reduced by about 20% when the substitution ratio of slag powder and fly ash is 30% and 20%, respectively. While the production cost of CCM is RMB 200/t, the production cost of LGM is about RMB 160/t. According to reference [33], the service life of LGM can be extended by about 20%, which significantly reduces the long-term maintenance cost and indicates that LGM is more economical in long-term use.

## 5. Conclusion

The study experimentally analyzed the strength, durability, and microstructural properties of LGCM and evaluated its economic and environmental benefits. The following are the key findings and conclusions of this study:

(1) Strength and durability: the LGCM outperformed the CCM in terms of compressive, flexural, and TS.
(2) SEM analysis revealed that the microstructure of the LGCM was denser and less porous, and this type of structure helped to improve the resistance of the LGCM to carbonation, infiltration, and sulfate attack.
(3) LGCM is better than CCM in terms of raw material cost, production cost, and long-term maintenance cost. In addition, its carbon emissions from LCA are lower than those of CCM, renewable energy cement, and carbon capture and storage technology, which have positive environmental benefits.

In summary, LGCM has demonstrated remarkable performance in terms of strength, durability, and environmental benefits, with a wide range of potential applications. It is recommended that LGCM be prioritized in scenarios where durability and low maintenance costs are paramount. Furthermore, the study suggests that government entities and relevant organizations consider implementing policies to encourage the adoption of LGCM in the construction industry, with the aim of promoting the sustainable development of this sector. However, its practical application still faces some challenges that require further research and optimization.The performance of LGCM may vary under different climatic conditions, and in actual construction, the construction technology of LGCM needs to be further optimized. In the future, the study will further explore the application of LGCM in large-scale production and practical engineering, with a view to promoting the sustainable development of construction materials.

## Supporting information

S1 File Minimal data set definition. (DOCX)
