# Supplementary material for: Strength and durability enhancement of low carbon gel concrete
Source: PLoS One. 2025 Jun 3;20(6):e0324319. doi: 10.1371/journal.pone.0324319 (PMC12133175; doi:10.1371/journal.pone.0324319)
Supplement: S1 File — (DOCX) [file pone.0324319.s001.docx]

**Table 1 The minimal data set for Figure 1**

| Water reducing agent dosage/% | Fluidity of clean slurry/mm | | |
| --- | --- | --- | --- |
|  | A-20 | A-32 | B cementitious material system |
| 0.02 | - | - | - |
| 0.04 | 140 | 125 | - |
| 0.06 | 230 | 150 | 155 |
| 0.08 | 270 | 250 | 245 |
| 0.10 | 300 | 295 | 300 |
| 0.12 | 300 | 300 | 305 |
| 0.14 | - |  | - |

**Table 2 The minimal data set for Figure 4**

| Time/d | Compressive strength/MPa | | | | | | | | | |
| --- | --- | --- | --- | --- | --- | --- | --- | --- | --- | --- |
|  | C30-J | C30-A-20 | C30-A-24 | C30-A28 | C30-A-32 | C50-J | C50-A-20 | C50-A-24 | C50-A-28 | C50-A-32 |
| 3 | 18.29 | 27.75 | 27.75 | 28.02 | 29.98 | 33.45 | 45.60 | 49.88 | 50.01 | 56.23 |
| 7 | 24.23 | 34.21 | 40.01 | 37.22 | 37.22 | 48.90 | 47.78 | 51.53 | 57.87 | 57.85 |
| 28 | 42.26 | 36.87 | 37.25 | 43.20 | 44.62 | 56.78 | 54.35 | 57.00 | 69.00 | 69.02 |

**Table 4 The minimal data set for Figure 5**

| (a) | | | (b) | | |
| --- | --- | --- | --- | --- | --- |
| Clinker ratio /% | Compressive strength/MPa | | Clinker ratio /% | Compressive strength/MPa | |
|  | A | B |  | A | B |
| 20 | 27.25 | 27.80 | 20 | 34.35 | 27.56 |
| 24 | 27.27 | 31.68 | 24 | 39.89 | 37.23 |
| 28 | 28.30 | 33.21 | 28 | 37.22 | 37.21 |
| 32 | 30.15 | 30.89 | 32 | 37.21 | 33.45 |
| (c) | | | (d) | | |
| Clinker ratio /% | Compressive strength/MPa | | Clinker ratio /% | Compressive strength/MPa | |
|  | A | B |  | A | B |
| 20 | 37.52 | 37.68 | 20 | 45.36 | 47.67 |
| 24 | 38.25 | 42.33 | 24 | 48.78 | 48.90 |
| 28 | 42.52 | 38.25 | 28 | 50.09 | 50.08 |
| 32 | 45.00 | 44.87 | 32 | 55.57 | 46.25 |
| (e) | | | (f) | | |
| Clinker ratio /% | Compressive strength/MPa | | Clinker ratio /% | Compressive strength/MPa | |
|  | A | B |  | A | B |
| 20 | 30.45 | 30.44 | 20 | 55.61 | 59.88 |
| 24 | 32.57 | 32.48 | 24 | 60.93 | 59.87 |
| 28 | 36.89 | 34.26 | 28 | 68.27 | 65.43 |
| 32 | 35.98 | 31.89 | 32 | 69.75 | 60.01 |

**Table 5 The minimal data set for Figure 6**

| Age (d) | Compressive strength (Mpa) | | | | | | | | | |
| --- | --- | --- | --- | --- | --- | --- | --- | --- | --- | --- |
|  | C30 | | | | | C50 | | | | |
|  | J | A-24 | A-32 | B-24 | B-32 | J | A-24 | A-32 | B-24 | B-32 |
| 0 | 17.25±0.11 | 25.00±0.03 | 24.37±0.02 | 25.23±0.12 | 25.21±0.08 | 32.21±0.07 | 43.24±0.21 | 43.20±0.11 | 43.21±0.09 | 43.12±0.20 |
| 20 | 20.23±0.12 | 22.67±0.21 | 21.13±0.62 | 23.01±0.04 | 22.00±0.12 | 56.78±0.23 | 60.12±0.11 | 57.89±0.23 | 60.01±0.12 | 58.47±0.11 |
| 40 | 25.46±0.31 | 27.42±0.38 | 26.78±0.01 | 28.45±0.41 | 27.33±0.41 | 62.32±0.12 | 67.25±0.91 | 63.08±0.13 | 65.79±0.31 | 65.78±0.21 |
| 60 | 30.89±0.91 | 32.91±0.19 | 31.93±0.14 | 33.56±0.31 | 32.58±0.32 | 63.34±0.12 | 67.57±0.14 | 63.98±0.21 | 65.88±0.23 | 66.01±0.03 |
| 80 | 35.56±0.12 | 37.24±0.49 | 36.47±0.34 | 38.46±0.41 | 37.48±0.56 | 64.89±0.04 | 71.23±0.23 | 66.22±0.25 | 68.25±0.42 | 71.30±0.01 |
| 100 | 40.89±0.21 | 42.44±0.01 | 41.77±0.31 | 43.00±0.42 | 42.59±0.42 | 65.88±0.32 | 72.98±0.21 | 66.78±0.08 | 70.23±0.05 | 73.00±0.03 |
| 120 | 43.87±0.45 | 45.34±0.90 | 44.56±0.34 | 46.24±0.91 | 45.57±0.41 | 66.03±0.48 | 73.21±0.23 | 67.78±0.89 | 70.98±0.42 | 73.24±0.05 |
| 140 | 45.03±0.21 | 47.28±0.04 | 46.98±0.58 | 48±0.31 | 47.55±0.41 | 67.25±0.42 | 73.45±0.66 | 68.23±0.12 | 71.03±0.05 | 73.45±0.12 |
| 160 | 47.87±0.13 | 49.89±0.04 | 48.89±0.13 | 50.54±0.35 | 49.52±0.45 | 68.77±0.36 | 73.67±0.11 | 70.57±0.05 | 71.10±0.06 | 73.21±0.09 |
| 180 | 52.12±0.09 | 51.52±0.81 | 48.88±0.49 | 51.14±0.41 | 53.57±0.41 | 70.53±0.34 | 74.03±0.09 | 71.14±0.06 | 71.16±0.10 | 72.14±0.24 |

**Table 6 The minimal data set for Figure 7**

| CM | | Flexural strength/MPa | Tensile strength/MPa |
| --- | --- | --- | --- |
| C30 | J | 4.82±0.23 | 2.67±0.05 |
|  | A-24 | 5.82±0.05 | 3.25±0.21 |
|  | A-32 | 5.04±0.31 | 2.78±0.13 |
|  | B-24 | 5.27±0.07 | 3.13±0.05 |
|  | B32 | 5.22±0.02 | 3.04±0.02 |
| C50 | J | 6.34±0.21 | 4.10±0.05 |
|  | A-24 | 7.62±0.05 | 4.87±0.01 |
|  | A-32 | 6.87±0.32 | 4.37±0.03 |
|  | B-24 | 7.55±0.24 | 4.69±0.04 |
|  | B-32 | 7.38±0.09 | 4.58±0.01 |

**Table 7 The minimal data set for Figure 9**

| (a) | | | | | |
| --- | --- | --- | --- | --- | --- |
| Curing time (d) | J | A-24 | A-32 | B-24 | B-32 |
| 0 | 1.7 | 1.3 | 2.7 | 1.8 | 2.2 |
| 1 | 1.5 | 1.2 | 1.6 | 1.2 | 1.5 |
| 3 | 1.4 | 1.0 | 1.0 | 1.1 | 1.2 |
| 7 | 1.2 | 0.8 | 1.1 | 1.0 | 0.9 |
| (b) | | | | | |
| Curing time (d) | J | A-24 | A-32 | B-24 | B-32 |
| 0 | 3,9 | 2.7 | 4.9 | 4.0 | 4.1 |
| 1 | 3.1 | 2.6 | 3.5 | 2.6 | 2.9 |
| 3 | 3.0 | 2.8 | 2.9 | 2.5 | 2.9 |
| 7 | 1.5 | 1.7 | 1.5 | 1.5 | 1.7 |
| (c) | | | | | |
| Curing time (d) | J | A-24 | A-32 | B-24 | B-32 |
| 0 | 4.0 | 3.5 | 5.1 | 4.7 | 4.6 |
| 1 | 3.3 | 3.5 | 3.5 | 3.2 | 3.7 |
| 3 | 3.6 | 2.7 | 3.6 | 3.4 | 3.5 |
| 7 | 3.5 | 2.2 | 3.0 | 2.5 | 3.0 |
| (d) | | | | | |
| Curing time (d) | J | A-24 | A-32 | B-24 | B-32 |
| 0 | 2.0 | 0.7 | 1.3 | 1.0 | 1.6 |
| 1 | 1.0 | 0.2 | 0.9 | 1.1 | 1.5 |
| 3 | 0.7 | 0.1 | 0.9 | 1.0 | 1.2 |
| 7 | 0.4 | 0.2 | 0.2 | 0.4 | 0.7 |
| (e) | | | | | |
| Curing time (d) | J | A-24 | A-32 | B-24 | B-32 |
| 0 | 2.3 | 1.2 | 1.8 | 2.4 | 2.8 |
| 1 | 2.4 | 0.6 | 1.1 | 2.1 | 2.0 |
| 3 | 0.9 | 0.2 | 1.1 | 1.1 | 1.2 |
| 7 | 0.9 | 0.2 | 0.6 | 1.3 | 1.1 |
| (f) | | | | | |
| Curing time (d) | J | A-24 | A-32 | B-24 | B-32 |
| 0 | 2.5 | 1.0 | 1.5 | 2.3 | 2.4 |
| 1 | 1.7 | 1.0 | 1.2 | 1.6 | 2.0 |
| 3 | 1.4 | 0.6 | 1.0 | 1.4 | 1.5 |
| 7 | 1.3 | 0.4 | 0.6 | 1.0 | 1.4 |

**Table 8 The minimal data set for Figure 10**

| CO_2_ concentration/% | 0d | 7d | 14d | 28d | Depth/mm | 1% | 3% | 5% | 10% | 20% |
| --- | --- | --- | --- | --- | --- | --- | --- | --- | --- | --- |
| 0 | 0.0 | 1.4 | 1.8 | 2.1 | 0 | 8.7 | 8.7 | 8.7 | 8.7 | 8.7 |
| 5 | 2.2 | 4.7 | 7.3 | 8.8 | 10 | 12.7 | 12.7 | 10.5 | 9.5 | 9.5 |
| 10 | 7.5 | 13.2 | 16.8 | 18.4 | 20 | - | - | 12.7 | 12.0 | 9.8 |
| 15 | 8.2 | 15.7 | 17.5 | 21.1 | 30 | - | - | - | 12.7 | 12.7 |
| 20 | 10.4 | 16.1 | 20.9 | 22.5 | 40 | - | - | - | - | - |

**Table 9 The minimal data set for Figure 11**

| K/% | C30 | | | | | C50 | | | | |
| --- | --- | --- | --- | --- | --- | --- | --- | --- | --- | --- |
|  | J | A-24 | A-32 | B-24 | B-32 | J | A-24 | A-32 | B-24 | B-32 |
| 30 | 102.5 | 105.4 | 102.3 | 100.1 | 105.2 | 102.5 | 106.2 | 101.1 | 102.5 | 102.0 |
| 60 | 106.7 | 109.2 | 107.0 | 107.0 | 107.8 | 108.9 | 107.4 | 104.6 | 102.9 | 107.0 |
| 90 | 107.5 | 113.5 | 109.5 | 108.6 | 111.5 | 111.3 | 108.9 | 106.2 | 105.8 | 108.4 |
| 120 | 102.5 | 112.3 | 104.2 | 104.2 | 104.2 | 106.2 | 105.4 | 104.2 | 105.7 | 105.3 |
| 150 | 97.5 | 108.7 | 98.7 | 102.5 | 98.1 | 103.4 | 95.1 | 98.7 | 103.4 | 95.8 |
| 180 | 95.8 | 106.8 | 96.2 | 97.8 | 95.8 | 97.1 | 88.4 | 95.8 | 101.6 | 92.1 |

**Table 10 The minimal data set for Figure 12**

| Cycles | C30 | | | | | C50 | | | | |
| --- | --- | --- | --- | --- | --- | --- | --- | --- | --- | --- |
|  | J | A-24 | A-32 | B-24 | B-32 | J | A-24 | A-32 | B-24 | B-32 |
| 0 | 100 | 100 | 100 | 100 | 100 | 100 | 100 | 100 | 100 | 100 |
| 50 | 98 | 97 | 96 | 99 | 97 | 97 | 99 | 97 | 97 | 99 |
| 100 | 83 | 93 | 93 | 98 | 93 | 97 | 98 | 97 | 97 | 98 |
| 150 | 76 | 81 | 88 | 93 | 90 | 96 | 97 | 95 | 93 | 96 |
| 200 | 64 | 81 | 79 | 93 | 64 | 96 | 95 | 94 | 95 | 93 |
| 250 | 33 | 70 | 68 | 71 | 58 | 78 | 95 | 92 | 92 | 83 |
| 300 | 27 | 64 | 60 | 60 | 46 | 70 | 93 | 86 | 88 | 78 |
| 350 | - | - | - | - | - | 59 | 75 | 74 | 78 | 65 |
